# How Social and Nonsocial Context Affects Stay/Leave Decision-Making: The Influence of Actual and Expected Rewards

**DOI:** 10.1371/journal.pone.0135226

**Published:** 2015-08-07

**Authors:** Amber Heijne, Alan G. Sanfey

**Affiliations:** 1 Department of Cognitive Science and Education, University of Trento, Trento, Italy; 2 Donders Centre for Cognitive Neuroimaging, Radboud University, Nijmegen, the Netherlands; 3 Behavioral Science Institute, Radboud University, Nijmegen, the Netherlands; Brain and Spine Institute (ICM), FRANCE

## Abstract

This study investigated whether deciding to either stay with or leave a social relationship partner, based on a sequence of collaborative social interactions, is impacted by (1) observed and (2) anticipated gains and losses associated with the collaboration; and, importantly, (3) whether these effects differ between social and nonsocial contexts. In the social context, participants played an iterated collaborative economic game in which they were dependent on the successes and failures of a game partner in order to increase their monetary payoff, and in which they were free to stop collaborating with this partner whenever they chose. In Study 1, we manipulated the actual success rate of partners, and demonstrated that participants decided to stay longer with 'better' partners. In Study 2, we induced prior expectations about specific partners, while keeping the objective performance of all partners equal, and found that participants decided to stay longer with partners whom they expected to be 'better' than others, irrespective of actual performance. Importantly, both Study 1 and 2 included a nonsocial control condition that was probabilistically identical to the social conditions. All findings were replicated in nonsocial context, but results demonstrated that the effect of prior beliefs on stay/leave decision-making was much less pronounced in a social than a nonsocial context.

## Introduction

People typically group together: they work together, become friends, join sports teams, and fall in love. However, as is evident from daily life, these social relationships we form with others do not always function the way we would like. That is, collaborations with colleagues turn out to be inefficient, friends let us down, we lose important games with our team, and we fight with our romantic partners. As a consequence, people are at some point faced with the decision to either maintain or terminate a social relationship.

Deciding to stay with or leave a social partner is one of the more complex social decisions a person must make. Numerous aspects feed into the decision process [1], such as individual factors (e.g., personality traits), relationship factors (e.g., investment in, and satisfaction with, a partnership), or even external factors (e.g., the influence of social networks). Additionally, the decision is often made in highly uncertain circumstances, as the decision-maker usually does not know the precise future consequences of staying or leaving. We propose that, in general, people will be more likely to stay in relationships the more they get out of them; and as people cannot know what a relationship will yield in the future, we additionally propose that people are also more likely to stay in relationships the higher the *expected* value of the relationship is, to some extent irrespective of the actual gains and losses.

Previous research supports the idea that people stay longer in relationships that provide them with more. That is, based on the proposition that people try to maximize their returns from social relationships [2], research has consistently demonstrated that self-reported satisfaction with real-life social relationships (which may capture subjective returns of a relationship) is one of the most important predictors of (intended) relationship maintenance [3, 4]. The fact that these findings are based on different types of real-life relationships (e.g., personal and work relationships, and even nonsocial commitments) supports the ecological validity of the link between relationship returns and relationship maintenance. However, as experimental testing of the *causal* link between relationship returns and *actual* stay/leave decision-making is lacking, the first goal of this paper is to establish that stay/leave decision-making is indeed affected by the outcomes one gains from a social partnership.

A related set of work examines the circumstances under which participants stop cooperating with an interaction partner in iterated economic games such as the Prisoner’s Dilemma [5, 6]. However, this line of work has mostly concerned itself with the question whether including an option to exit, that is, to dissolve a partnership, affects cooperative or trusting decisions. In contrast, our goal here is not to investigate how the option of leaving a partner affects cooperation levels, but rather whether the collaborative success of a partnership affects subsequent decisions to either stay or leave.

Given that people are more likely to stay in social relationships the higher the relationship returns are, it makes intuitive sense that people will also be more likely to stay in social relationships the higher they *expect* the relationship returns to be. This also follows from the softmax selection rule in reinforcement learning [7], which predicts that a decision maker becomes more likely to select an action as the expected returns of that action get higher. Previous research demonstrates that prior expectations can indeed have strong effects on social decision making. For example, in the context of fairness, it has been shown that psychological descriptions about a specific partner [8] or information about what a typical partner would do [9] influence the rejection of unfair offers in the Ultimatum Game. Similarly, in the context of trust, it has been demonstrated that explicit descriptions of a person’s moral character [10], as well as implicit assessments of a social partner’s trustworthiness based on his or her facial features [11] influence how much trust a person puts in a social partner in the Trust Game. Even more, implicit race attitudes have also been found to influence social decision making in the contexts of fairness [12] and trust [13, 14]. Finally, people are more likely to cooperate with partners in a Prisoner’s Dilemma when they perceived these partners as cooperative based on a brief informal conversation beforehand [15]. The second goal of the present paper is therefore to investigate whether, and how, expectations about relationship returns affect decisions to stay in a social relationship. Specifically, we will focus on those situations in which decision makers have exaggeratedly high or low prior expectations about a partner.

An important question here is *how* expectations might affect decisions to stay with or leave a social partner. One possibility is that prior expectations are used as a frame of reference against which actual returns of a partnership are compared. In this way, overly low expectations about an average partnership could lead to the experience that actual returns are better than expected (i.e., positive prediction error) which, in turn, could have a *positive* effect on the length of time people decide to stay in a partnership. In contrast, having overly high expectations about the same average partnership could induce the experience that actual returns are *worse* than expected (i.e., negative prediction error) which would have a *negative* effect on the time people decide to stay. Thus, one prediction could be that people will stay longer in an average partnership if they had relatively low prior expectations, and shorter if they had high prior expectations about the partnership. This way of updating expectations with actual observations is typical for associative learning [7].

However, previous research demonstrates that having prior beliefs about decision options can impair associative learning, such that people tend to retain prior beliefs rather than updating them with new observations. In this way, the presence of prior beliefs can even harm decision making when those prior beliefs are incorrect[16, 17]. To illustrate, several studies have demonstrated that people are likely to trust interaction partners when they initially believed these partners to be trustworthy even if these partners, in reality, did not reciprocate their trust [10, 18]. If this holds also for stay/leave decision making, then we would formulate an opposing set of predictions regarding the effects of prior expectations. Specifically, we would predict that people would stay shorter in an average partnership if they had low prior expectations about the partnership; and longer if they had high prior expectations about the partnership.

The two theoretical proposals outlined above as to how prior expectations could affect stay/leave decision making lead to the formulation of two opposing sets of predictions. The empirical question addressed here is therefore exactly how prior expectations impact people's decisions to either stay with or leave a social partner.

Although we propose rather deliberative, or even “coldhearted”, mechanisms underlying decisions to stay with or leave a social partner, it seems clear that choosing to leave a social partner may have important differences than abandoning a monetary investment that is not paying off. We expect additional mechanisms to be at play when deciding about social partners as opposed to a monetary, or other non-social, resource. For example, when a social relationship is not productive, underlying feelings of bonding, or perhaps guilt, may prevent someone from leaving the social partnership even though this may be the optimal decision in terms of payoffs. Indeed, feelings of social bonding have been reported even in experiments in which participants merely had to synchronize finger tapping [19], and it has been theorized that people are intrinsically motivated to maintain social connections and resist breaking them [20]. These feelings of bonding, or intrinsic motivations to stay, presumably do not occur when deciding about a purely probabilistic resource, but they could well impact stay/leave choices about social relationship partners. The third goal of this paper is therefore to directly compare effects of anticipated and observed gains and losses on stay/leave decision making between social and nonsocial contexts; with an associated prediction that people would have specific biases towards stay decisions in a social context.

To explore these questions, we employ a novel task, the Apple Game, to simulate real social relationships between participants in the lab. In the Apple Game, a cooperative relationship is formed between two players, the participant and another, anonymous, partner. We use a free-choice paradigm to increase ecological validity, with the participant free to leave their game partner at any moment, at which point they are paired with another partner. Moreover, the Apple Game has also the important cooperative characteristic that participants share a mutual goal, with no benefit in exploiting the other. We note this explicitly, as this is not the case in the standard tasks that simulate cooperative relationships between players. For example, to achieve the optimal personal economic outcome, players should decide to free-ride in Public Good games; defect in the Prisoner’s Dilemma; not reciprocate trust in the Trust Game; or make the lowest possible offer that still has a likelihood of being accepted in the Ultimatum Game. In contrast, in the Apple Game, to obtain the highest (personal) income, participants are required to cooperate, and failures of cooperation will negatively impact the income of both players.

In summary, in Study 1, we test the hypotheses that (1a) people decide to stay longer in collaborative partnerships as the probability of success with that partner rises, (1b) that people stay longer with non-social resources with a higher probability of success, and additionally, whether people are biased towards staying rather than leaving in a social context specifically. Study 2 examines whether, and how, prior expectations about partnership returns impact stay/leave decisions; and we additionally explore how social versus nonsocial context impacts the effect of prior expectations on stay/leave decision making.

## Study 1

In Study 1, we investigate the hypothesis that people try to maximize the value they obtain from their relationships by staying in partnerships with a high success rate, and leaving partnerships with a low success rate. Furthermore, we directly compare this effect between social and nonsocial contexts, to investigate whether the actual observed gains and losses experienced in a social collaboration affect stay/leave decision making to the same extent as in nonsocial contexts.

## Materials & Methods

### Participants

One-hundred and seventeen students (mean age = 19.6, 87% female) from Radboud University in Nijmegen, The Netherlands, were recruited via an online participant database. Participants played in return for course credit, and were additionally incentivized to perform on the task by the allotment of a performance dependent monetary bonus to six randomly selected participants (7.60 Euros on average). Ethical approval is provided by the responsible local ethics committee CMO regio Arnhem-Nijmegen (i.e. acknowledged Dutch Review Board).

### Procedure

**Fig 1:**
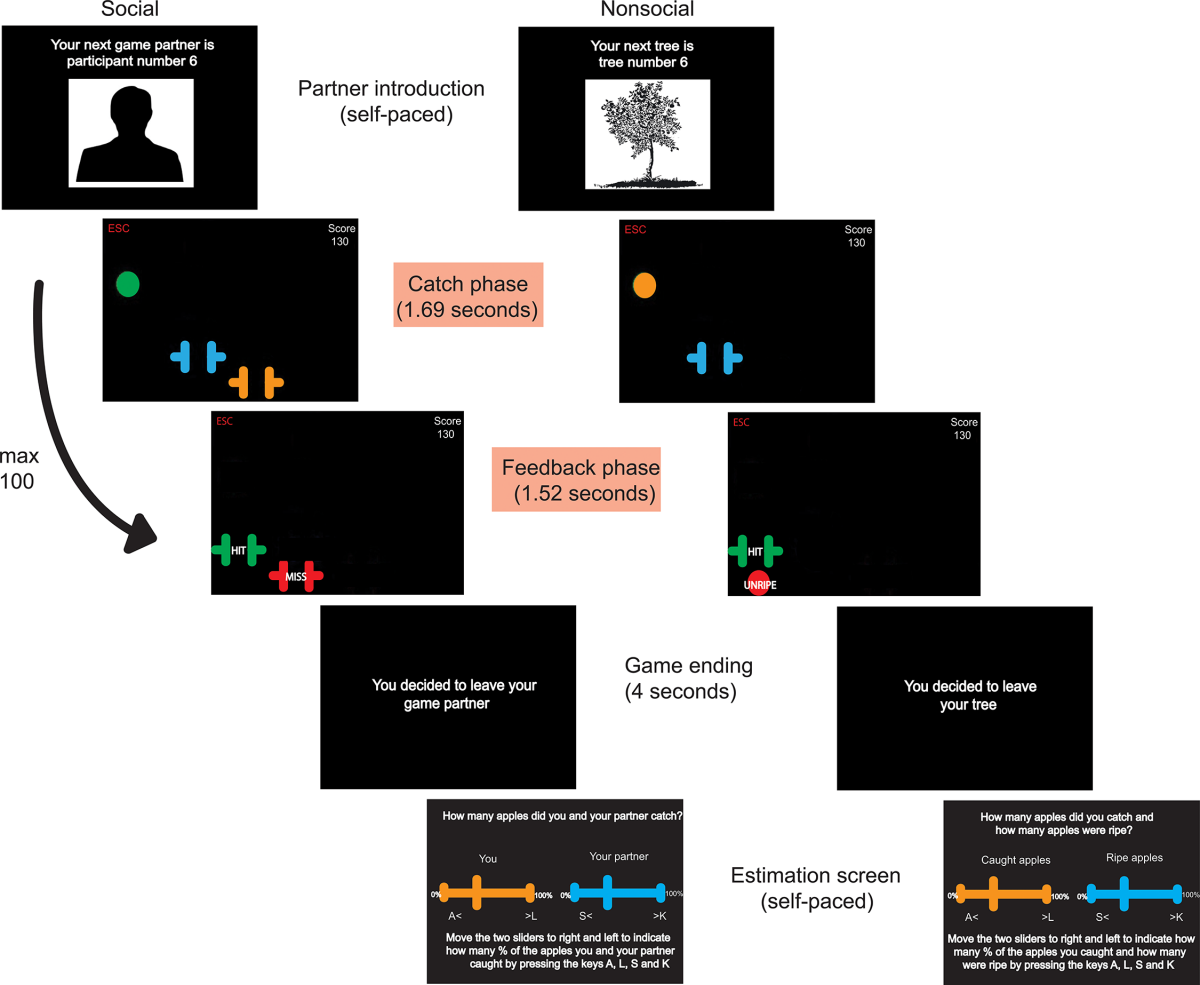
Timeline of the Apple Game. Participants played the Apple Game with multiple options that they encountered in sequential order. Each sub-game began with an introduction screen on which the option was introduced by a silhouette and arbitrary ID-number. Once participants pressed the space bar, the apples immediately started falling. Each trial consisted of a catch (1.69 seconds) and feedback phase (1.52) seconds. Trials automatically and immediately followed one another. When participants pressed the ESC button, or when the maximum number of trials (i.e., one-hundred) was reached, a game was stopped. Participants were then asked to estimate their own and their option's performance and were introduced to a new option. This cycle repeated itself until the total number (650) of apples had fallen.

After receiving written instructions about the task (see “S1 Appendix” and “S2 Appendix”), participants started playing the Apple Game, which lasted for forty-five minutes (Fig 1). Half of the participants played a social version of this game, and the other half played a nonsocial version. In both versions, the participant’s task was to collect as many points as possible by catching virtual apples that fell from the top of the computer screen. Specifically, at the start of each of the 650 trials, an apple appeared at a random horizontal position at the top of the screen, and immediately started falling. At the same time, a virtual basket was displayed on the screen that could be moved in a horizontal (left/right) direction by the participant (by pressing the ‘L’ and ‘A’ keys on the keyboard). The participant’s task was to move their basket under the falling apple in time, such that the apple would fall through the basket.

Apples fell quite quickly (i.e., it took 1.69 seconds for an apple to fall from the top to the bottom of the screen). After the apple reached the bottom of the screen, participants briefly (i.e., 1.52 seconds) received feedback about whether they caught the apple (the basket turning green and displaying the word ‘hit’) or not (the basket turning red and displaying the word ‘miss’). Each trial thus consisted of a catch and feedback phase. After each trial, the number of points the participant collected were immediately updated and displayed in the top right corner of the screen. The specific timing intervals used for the catch and feedback phase of each trial were calculated based on the refresh rates of the computer monitor to ensure the graphics were smooth.

A key feature of the Apple Game was that participants' outcomes were not only determined by their own 'hits' and 'misses' but also by the successes and failures of an external source. The nature of this external source depended on whether participants played the social or nonsocial version of the game. That is, participants could either play the game with, ostensibly, another participant (hereafter called the partner) who also tried to catch the apples. In this case, participants' outcomes were affected by catches or misses of the partner (i.e., the social condition). In the other condition, outcomes were affected by whether a caught apple had been ripe or not (i.e., the nonsocial condition).

**Fig 2:**
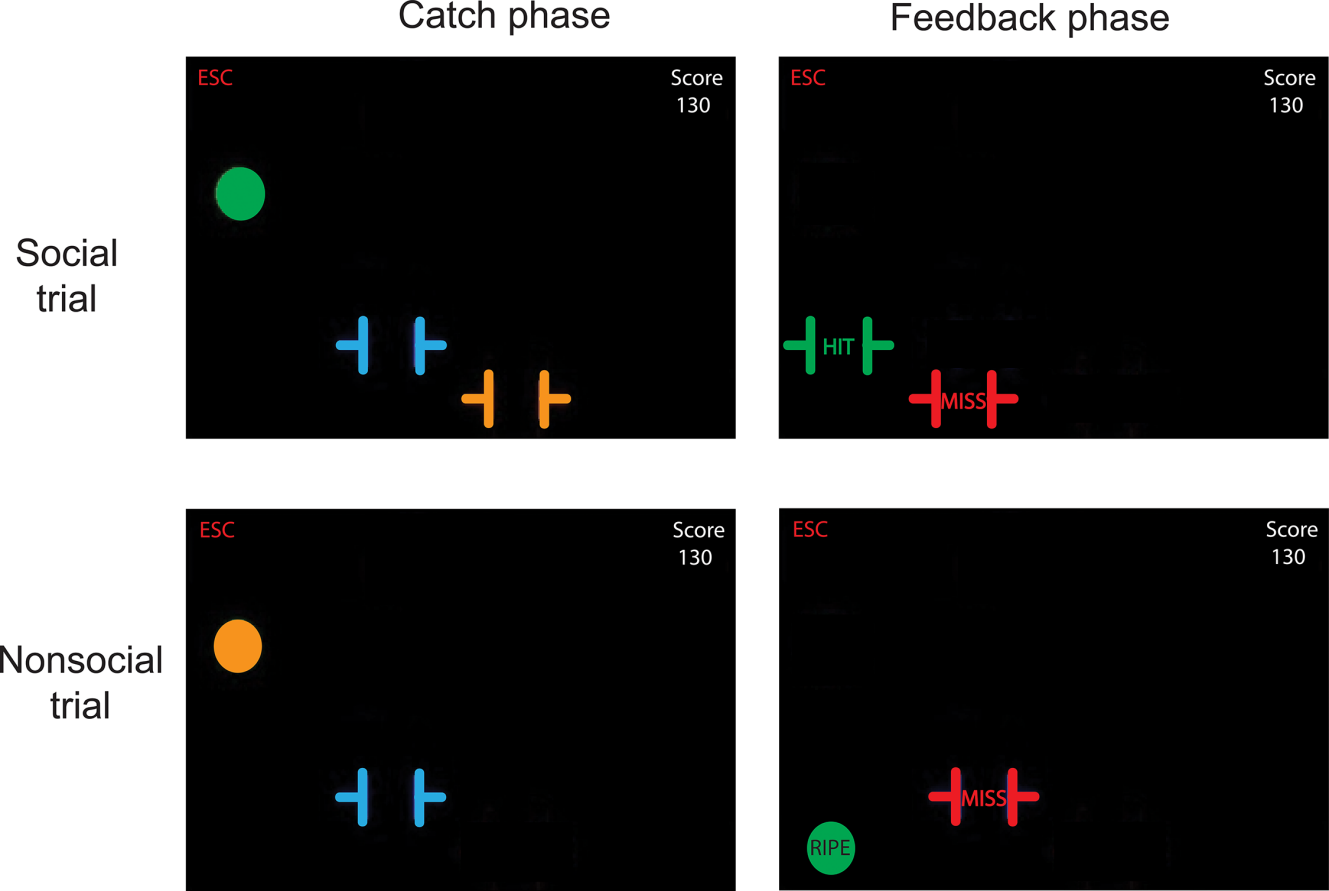
Catch and feedback phase of each trial in social and nonsocial task version. On each trial, an apple fell. In the social task version, both participant and co-player tried to catch the apple and, immediately after, feedback was given as to who caught the apple, and who did not. In the nonsocial task version, the participant tried to catch the apple. After the apple reached the bottom of the screen, participants learned whether they caught the apple or not and whether the apple had been ripe.

More specifically, in the social condition, participants were paired up with a partner whose task was also to catch apples. During the catch phase of each trial, participants saw also a second virtual basket moving across the screen that was, ostensibly, controlled by the partner. In addition, during the feedback phase the partner's basket would also turn green or red and display the words 'hit' or 'miss' depending on whether the partner caught the apple or not (Fig 2). Importantly, as the social version of the Apple Game was intended to simulate collaborative relationships between participants and partners, they had to both perform well on this task to both earn points. That is, only apples that were caught by *both* of the participants led to a reward (ten points awarded to both participants), whereas failure to catch the apple by either one (or both) of the participants led to punishment (i.e., five points are removed from *both* participants’ scores). Note that this entails that a participant who catches many apples could still be losing points if they happened to be assigned to a poorly-performing partner.

In the nonsocial version, participants were assigned to trees that could drop either ripe or unripe apples (i.e., the equivalent of hits and misses by the co-player in the social version). To match the feedback and outcomes of the social task version, participants did not know whether an apple was ripe (i.e., it had an ambiguous orange color) during the catch phase. During the feedback phase however, ripe apples turned green and displayed the word 'ripe' whereas unripe apples turned red and displayed the word 'unripe' (Fig 2). Only the catching of ripe apples led to reward (ten points awarded to the participant), whereas either catching an unripe apple or the failure to catch an apple led to punishment (five points removed from the participant’s score). Similarly to the social version, a participant who managed to catch many apples could still be losing points if they had been assigned to a tree that dropped a large percentage of unripe apples.

Participants' outcomes were thus dependent on the successes and failures of their partner or tree (hereafter called an 'option'). But, and importantly so, participants had the possibility to leave an option, in which case they were assigned to a new—and potentially better—option. A leaving cue (i.e., the letters ESC in red) was continuously displayed in the top left corner of the screen; and to leave, participants simply pressed the ESC button on the keyboard. Participants were free to leave whenever and as often as they desired, with no associated cost. In addition, in the social condition specifically, participants were instructed that they could leave their partners but that their partners could not leave them. This was done to ensure that participants only decided to leave due to their dissatisfaction with a partner—and not because they, for example, feared that they themselves would be left by their partners.

Each time a partnership ended, participants answered several questions about their former option before being assigned to a new option. That is, participants were asked to estimate, and indicate on a slider, the percentage of the apples they themselves caught (in both social and nonsocial condition); and the percentage of apples their former partner had caught (social condition) or the percentage of ripe apples the former tree had dropped (nonsocial condition). After answering these questions, a new option was immediately introduced by means of a silhouette, along with an arbitrary ID-number.

The primary within-subject variable in Study 1 was the success rate (i.e., performance level) of options which were set to low, moderate or high. Specifically, partners would either catch 85% (i.e., high performance condition), 50% (i.e., moderate performance condition) or 15% (i.e., low performance condition) of the apples. Similarly, trees would drop 85% (i.e., high), 50% (i.e., moderate) or 15% (i.e., low) of ripe apples. Performance level was ordered pseudo-randomly with each of the three levels occurring twice in every sequence of six options. In addition, to ensure participants played at least twice with an option from each performance level across the experiment, participants were forced to leave after each 100 trials with a specific partner or tree (i.e., forced leaves). Finally, to ensure participants believed they were playing against actual other participants in the social version of this task, we modeled the movement of the partners’ baskets on the movements of a real player who played the game during the development phase of the task. Also, we invited multiple participants to the lab simultaneously to enhance participants’ belief that there were other players playing the game at the same time. Verbal debriefing of pilot participants and a random selection of the actual participants revealed no indication that participants did not believe the social manipulation.

## Results

### Descriptives

Across the entire course of the experiment, participants on average freely decided to leave their partners 6.67 (SD = 3.80) times and were forced to leave 5.41 (SD = 0.94) times. As a result, they played, on average, with 12.09 (SD = 3.82) partners in total. In the nonsocial condition, participants decided to leave 5.61 (SD = 3.10) times, and were forced to leave 4.38 (SD = 0.88) times. In the nonsocial condition, they played with 10.00 (SD = 2.99) different trees over the entire experiment. Note that the final partner or tree that participants played with was not included in the analyses, as this ‘leave’ decision was determined by the end of the experiment.

Staying times for different options varied considerably, even within participants. On average, participants stayed 60.96 rounds, with a minimum of 13.90 rounds and a maximum of 100 rounds. The average difference between the shortest and longest staying time within participants was 86.10 rounds. Between participants, differences in stay/leave decision making were also quite large, with some participants never making a leave decision (n = 3)–and thus playing with only 6 different options–while others decided to leave much more frequently and therefore interacted with up to 22 different options.

Interestingly, participants appeared to adjust their own performance to the performance of partners and trees. That is, a mixed model analysis demonstrated that the percentage of apples caught by a participant (i.e., the dependent variable) was significantly affected by the performance level of the various options (*F* = 29.92, *p* < .001) and this effect was significantly different between social and nonsocial task version (*F* = 3.82, *p =* .*024*). Participants’ own performance was, overall, not significantly different (*F* = 1.95, *p* = .116) between the social and nonsocial versions.

In the social condition, participants caught, on average, 56.38% (SD = 5.89), 52.44% (SD = 12.10), and 47.28% (SD = 17.80) of the apples when interacting with the high, moderate, and low performance partners respectively. Tukey's HSD post-hoc tests demonstrated that the difference in participants' performance when playing with high versus moderate performing (z = 3.80, p = .002) and moderate versus low performing partners (z = 5.16, p < .001) were both significant. In the nonsocial condition, participants caught approximately the same percentage of apples (z = -0.47, p = .997) when playing with trees with a high (M = 52.44%, SD = 9.46) and moderate (M = 53.25, SD = 9.19) performance level; but they caught significantly less apples (z = 6.19, p < .001) when playing with trees with a low as compared to a moderate performance level.

### Stay-Leave decisions

#### The model

In the present study, participants were free to decide to leave an option whenever they wanted. Therefore, we use the number of trials that they decided to stay with a given option as the dependent measure (i.e., staying time). However, there are two ways in which a partnership could be ended, namely when the participant pressed the ESC button or when the participant stayed for the maximum number of 100 trials. The latter ‘forced-leave’ event was implemented to ensure that participants interacted with options of all performance levels at least twice. In the current study, 4.92%, 27.05% and 68.02% of, partnerships with, low, moderate and high performing partners respectively ended in a forced leave in the social condition; and in the nonsocial condition 3.66%, 35.11% and 61.23% of partnerships with, respectively low, moderate and high performing trees ended in a forced leave. We treat these ‘forced-leave’ events as if the participant had decided to stay with an option for 100 trials. In our analyses, these events are thus not qualitatively different from the partnerships that were terminated by the participants’ themselves.

Since we have a mixed design with a highly unbalanced number of repeated measures per participant, the data were analyzed using a mixed-model approach. Specifically, we used the lmer function of the lme4 package in R [21]. F and p-values were computed using the mixed command within the afex package.

To investigate whether the number of stay trials was affected by performance levels, and whether this effect was moderated by task version, we constructed a linear mixed model (model 1A) using the number of stay trials as the continuous dependent measure, and performance level (high, moderate, or low; within-subject) and condition (social or nonsocial; between-subject) and their interaction term as fixed effects. We also included a number of variables that were not of primary interest but may have had an impact of choice, noted below.

Firstly, we observed previously that participants adjusted their own performance to the performance levels of different options. We therefore added to the model (a) the percentage of successful trials (i.e., trials on which participants received a reward); (b) the percentage of trials on which participants lost points due to their option's failure (i.e., a miss by a partner or an unripe apple dropped from a tree); and (c) percentage of trials on which participants lost points due to their own failure to catch an apple. Note that also adding the number of trials on which participants lost points due to both their own failure and the option's failure would make the model unidentifiable and, as such, this variable was not included in the model.

In addition, participants' estimations of the performance level of options may differ from the actual performance levels, and participants' ability to correctly estimate performance levels could even depend on the task version. Therefore, we also added to the model participants’ estimations of each options' performance and the interaction of this variable with task version.

Also, participants' staying times for low and moderately performing options may have decreased (a) the more they believed that there were better performing options 'out there' and (b) the better they became at identifying lower performing options. Both measures may increase with the number of high-performing options that participants had previously played with. Therefore, we also included the percentage of high (rather than low or moderate) performing options that a participant had already seen at the time of each decision. Finally, we included a fixed intercept as well as a participant-specific random adjustment to that intercept to account for the repeated measures design, and included random slopes for performance level to account for individual differences in the effect sizes for these factors. All continuous variables were z-scored in order to promote model convergence.

#### Results

**Fig 3:**
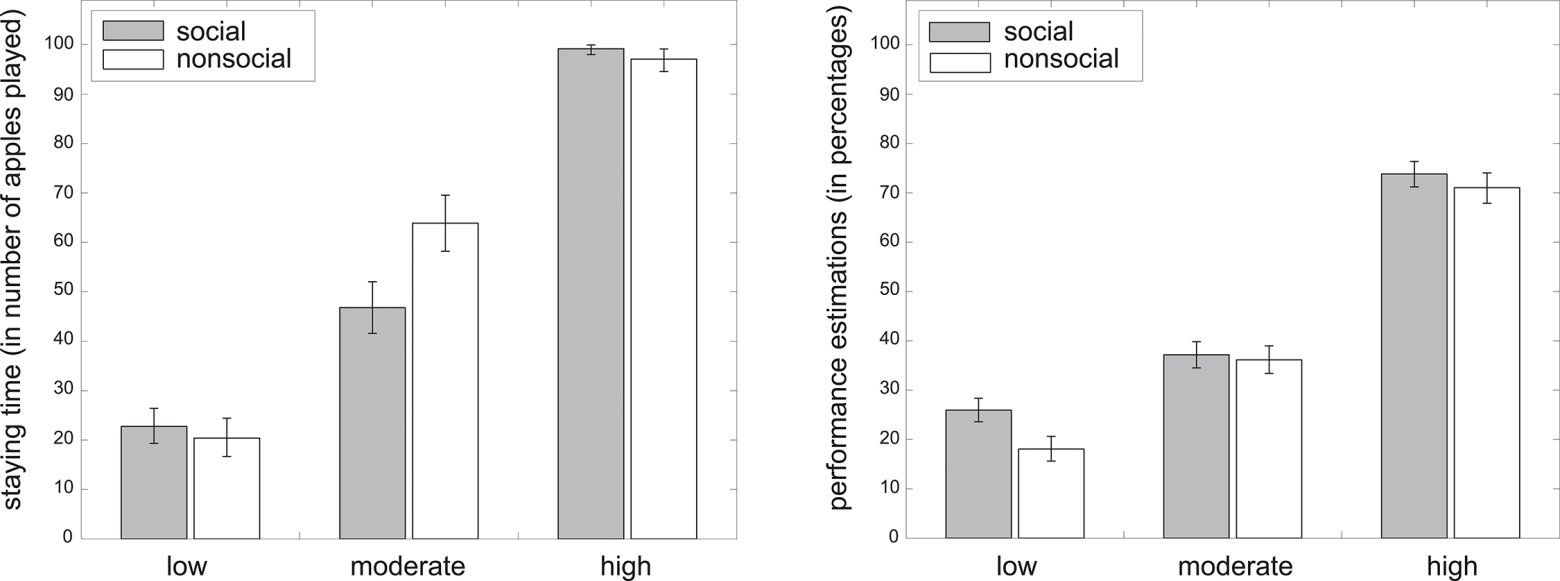
Staying times and performance estimations as a function of actual performance levels. Staying times (left graph) and performance estimations (right graph) for social (gray bars) and nonsocial (white bars) options that performed low (i.e.,15% success), moderately (i.e., 50% success) or high (i.e.,85% success) in the game. Error bars represent bootstrapped 95% confidence intervals.

Results demonstrated that the number of stay trials was significantly (*F* = 8.69, *p* < .001) affected by performance levels (participants stayed, on average, 21.83 rounds with low performing options, 46.77 rounds with moderately performing options and 98.35 rounds with high performing options, collapsed across social and nonsocial task version). The difference in staying times between low and moderately performing options (*t* = 31.87, *p* < .001) and the difference for moderately performing versus high performing options (*t* = 46.17, *p* < .001) are both significant. The main effect of task version on staying times (*F* = 2.77, *p* = .100) and the interaction effect of task version by performance level on staying times (*F* = 2.65, *p* = .070) are not significant (Fig 3).

In addition, participants stayed longer with options under the following circumstances: the higher they estimated the option’s performance (*F* = 40.07, *p* < .001, *β* = 0.25); the greater the percentage of trials on which participants' lost points due to their own failure to catch an apple (*F* = 31.11, *p* < .001, *β* = 0.59); the larger the percentage of trials on which participants gained points due to a mutual success (*F* = 124.60, *p* < .001, *β* = 1.48); and the fewer high-performing options they had previously seen (*F* = 60.19, *p* < .001, *β* = -0.30). The percentage of trials on which participants lost points due to an option's failure did not significantly impact staying times (*F* = 2.24, *p* = .130, *β* = 0.09); and the interaction effect of performance estimations by task version on staying times was also non significant (*F* = 0.27, *p* = .610).

### Estimations of performance

#### The model

To gain insight into how participants actually processed performance levels, we examined if participants were aware of the difference in performance between the different options, and whether the performance levels of options was evaluated in a similar way between social and nonsocial task versions. To test this, we set up a mixed model (model 1B) with performance estimations as the dependent measure; our performance manipulations (i.e., high, moderate, or low), task version (social or nonsocial) and the interaction term of these variables as fixed factors; and the percentage of high-performing options they had already paired up with; the percentage of trials on which participants' lost points due to their options' failure; the percentage of trials on which participants lost points due to their own failure; and the percentage of trials on which participants gained points. Again, both a fixed intercept and random participant-specific adjustment to the intercept, as well as random slopes for the within-subject factors were included to the model.

#### Results

The results show that participants indeed noticed the difference in performance between the different options, with a significant main effect of performance level on performance estimations (*F* = 82.07, *p* < .001). Participants believed the low, moderate and high performing options to perform at rates of 23.21%, 38.26% and 70.69% respectively. The differences in performance estimations between low- and moderate-performing options (*t* = 14.05, *p* < .001), as well as between moderate- and high-performing options (*t* = 35.87, *p* < .001) are significant. The main effect of task version on performance estimations was also significant (*F* = 4.34, *p* = .040), but the interaction effect (*F* = 1.86, *p* = .160) between the two factors was not (Fig 3). In addition, performance estimations significantly increased with the percentage of trials on which participants gained points due to mutual successes (*F* = 68.95, *p* < .001, *β* = 0.90). Performance estimations were not affected by the percentage of high-performing options already seen (*F* = 1.97, *p* = .160, *β* = 0.05); the percentage of trials on which participants lost points due solely to their own failure (*F* = 0.30, *p* = .580, *β* = -0.05) or solely their option's failure (*F* = 0.31, *p* = .580, *β* = -0.03).

### Mediation analysis

As a post-hoc analysis, we tested whether the effect of performance manipulations on staying times was mediated by participants’ estimations of their options’ performance level by inserting the output of model 1A and model 1B into the mediate function within the mediation package (Tingley et al., 2013). Results demonstrated that 7 of the total increase of 14.36 apples played with high- rather than moderate-performing options was mediated through participants' performance estimations (*p* = 0.00); and the remaining 7.36 apples were directly caused by the performance manipulations (*p* = .01). Furthermore, 6.67 of the total decrease of 11.26 apples that participants played with low- rather than moderate-performance partners were mediated through participants' performance estimations (*p* = .00); and the remaining 4.59 apples were directly caused by our performance manipulations, but this direct effect was not significant (*p* = .08). We can thus state that the effect of partner performance on staying times was partially mediated by participants’ estimations of their partners’ performance.

## Conclusion

Study 1 confirmed the initial hypothesis that people would decide to stay longer in partnerships the higher the actual returns of those partnerships were. Similar effects were found in both social and nonsocial conditions, and the effect of partner performance on staying times was partially mediated by participants’ estimations of their partners’ performance.

## Study 2

Study 2 was conducted to investigate how the interplay between anticipated and observed gains and losses associated with a social collaboration would affect stay/leave decision-making. Moreover, we compared these effects between social and nonsocial contexts to examine if expectations impact social and nonsocial partnerships to different extents.

## Materials & Methods

### Participants

Ninety-three students (mean age 20.2, 83% female) from Radboud University in Nijmegen, The Netherlands were recruited via an online database to participate in Study 2 in exchange for course credit. Five randomly selected participants additionally earned a performance-dependent monetary bonus, (7.52 Euros on average). Ethical approval was provided by local responsible ethics committee (as in Study 1).

### Procedure

**Fig 4:**
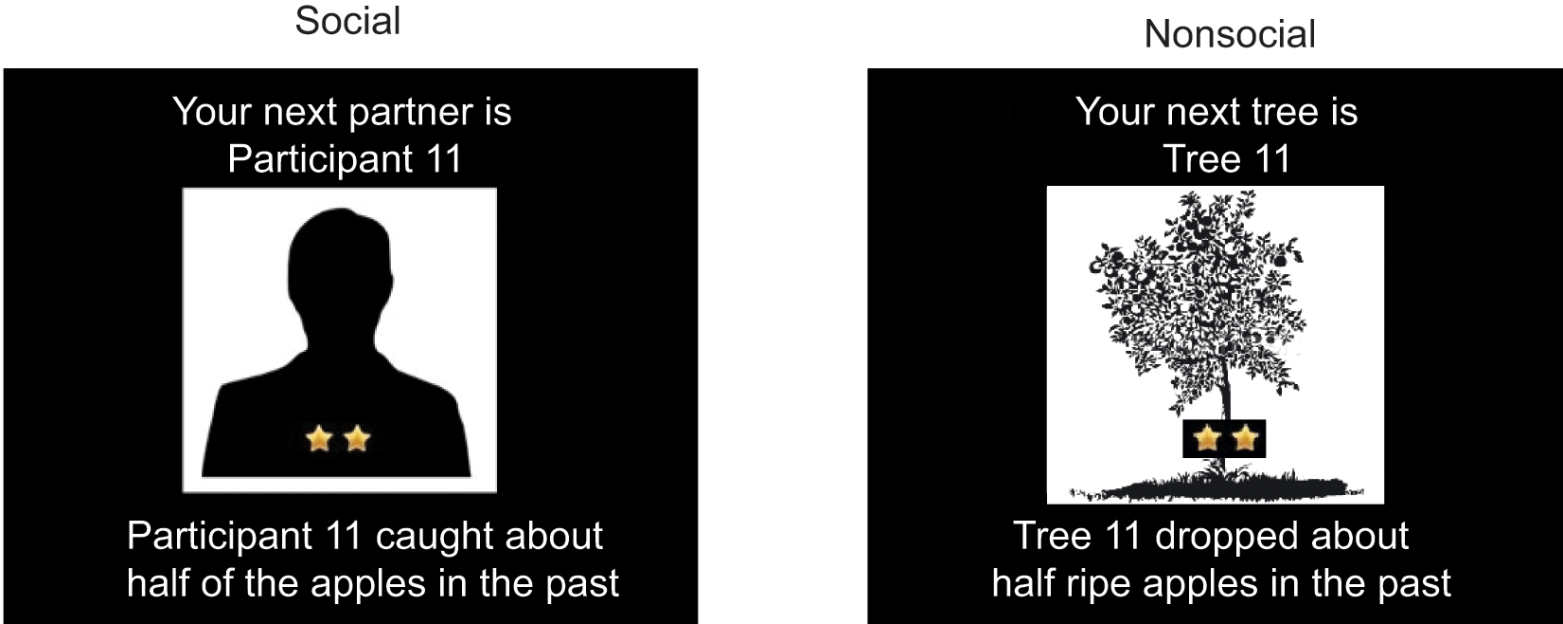
Screenshot, expectation induction. Introduction screen of a new partner in Study 2. The silhouette is coupled with an arbitrary ID number, a star rating, and written information about the participant’s past performance.

As in Study 1, half of the participants were randomly chosen to play the social version of the task (i.e. purportedly with another experimental participant), while the other half played the non-social version (i.e. with a tree). The game was as described in Study 1 (above), with the following differences: (1) the actual performance of all options in both social and nonsocial task version was now set at 50%, that is, there were no objective performance differences between the options; and (2) bogus information about the ostensible prior performance of each option was provided at the beginning of each partnership (Fig 4). Every time a new option was introduced to the participant, information about that option's past performance was provided via both a ‘star rating’ and text information. Options could have 1-, 2-, or 3-star rating, meaning that partners (in the social condition), caught less than half (i.e., 0–33%), about half (i.e., 34–66%) or more than half (i.e., 67%- 100%), respectively, of the apples in the past (Fig 4), and that trees (in the nonsocial condition), dropped less than half (i.e., 0–33%), about half (i.e., 34–66%), or more than half (i.e., 67–100%), respectively, of the apples in the past. During the instruction phase of the task (see “S1 Appendix” and “S2 Appendix”), participants were informed about the percentage rates for each category.

## Results

### Descriptives

Over the course of the experiment, participants decided to leave their co-players 6.62 (SD = 4.73) times, and were forced to leave them 3.91 (SD = 1.22) times. As such, they played, on average, with 10.53 (SD = 4.19) partners in total. In the nonsocial condition, participants freely left 6.91 (SD = 3.72) times; and were forced to leave 4.13 (SD = 1.02) times. As a result, they played with, on average, 11.04 (SD = 3.47) different trees. Participants' staying times varied considerably between options. The mean staying time was 57.22 rounds, and ranged between 14.86 rounds and 100 rounds. The average difference between the shortest and longest staying time within participants was 85.14 rounds.

Stay/leave decision-making also varied across participants, as observed in Study 1. Some participants never left their options (note that, as in Study 1, we did not include the final option in the analyses)–and thus played only with 6 different options over the course of the experiment—whereas others played, maximally, with 23 and 21 different social and nonsocial options respectively.

As in Study 1, it appeared that participants own performance adjusted to the perceived performance of their options. To test this, we built a mixed model analysis with the percentage of apples the participants themselves caught as the dependent variable; and expectations about their options (low, moderate, high) and condition (social, nonsocial) as the independent variables. In addition, we included a fixed intercept and a random participant-specific adjustment to that intercept; and a random slope for expectations to the model. The interaction effect of expectations by condition was significant (*F* = 15.65, *p* < .001) as were the main effects of expectations (*F* = 42.49, *p* < .001) and condition (*F* = 26.42, *p* < .001). Tukey's HSD post-hoc tests revealed that, in the social condition, participants caught significantly less (*z* = 3.28, *p* = .013) apples (53.67 percent, SD = 20.56) when playing with partners about whom they had low expectations compared to when they played with moderate expectancy partners (59.44 percent, SD = 11.39), but their own performance did not increase (*z* = 1.29, *p* = .782) when playing with high expectancy (61.68 percent, SD = 8.30) as compared to moderate expectancy partners. In the nonsocial condition, participants caught significantly more apples when playing with moderate (54.59 percent, SD = 18.54) rather than low (30.65 percent, SD = 33.73) expectancy trees; and again significantly (*z* = 3.97, *p* < .001) more apples when playing with high (61.49 percent, SD = 4.52) rather than moderate expectancy trees.

### Stay-Leave decisions

#### The model

Analyses were performed in a similar manner as described in Study 1. That is, we again used a mixed-model approach, using the lme4 package in R. The focus of this study was to investigate whether inducing prior expectations about specific options affected the stay/leave decisions made by participants, and whether this effect was different when playing the social or nonsocial task version. To investigate this, we built a linear mixed model (model 2A) with the number of stay trials as the continuous dependent measure, and with expectations (low, moderate or high; within-subject) and task version (social or nonsocial; between-subject) and their interaction term as fixed effects. Also, we took into account participants’ performance estimations and, as the ability to estimate performance could differ depending on the task version, the interaction of performance estimations with task version was also included. Furthermore, we included the percentage of high (rather than low or moderate) expectancy options a participant had previously interacted with; the percentage of trials on which participants lost points due to failures by their option; the percentage of trials on which participants lost points due to their own failure; and the percentage of trials on which participants gained points. In addition, as observations were nested within individual participants, we included a fixed intercept and a participant-specific random adjustment to that intercept to the model. Finally, we included random slopes for the within-subject factors expectations. All continuous variables were again z-scored.

#### Results

**Fig 5:**
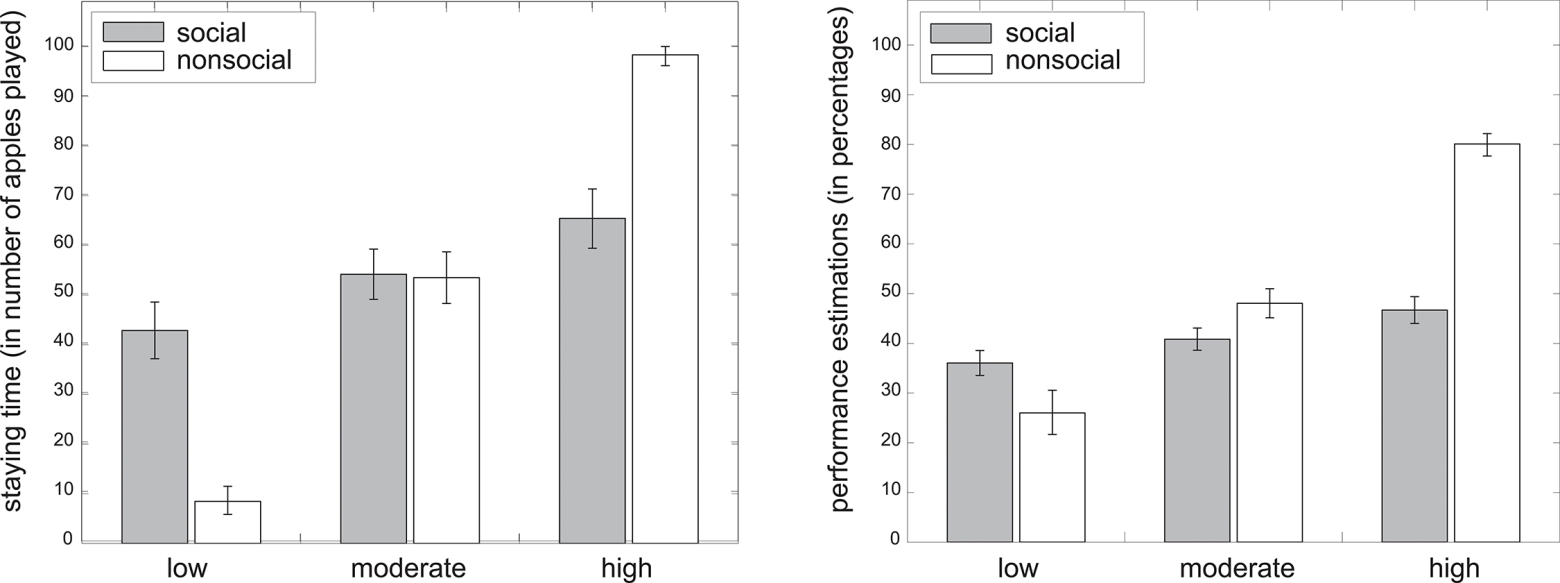
Staying times and performance estimations as a function of prior expectations. Staying times (left) and performance estimations (right) for social (gray bars) and nonsocial (white bars) options for whom participants had low, moderate and high prior expectations, (in reality all performed at a 50% success rate). Error bars represent bootstrapped 95% confidence intervals.

Results demonstrated that prior expectations about options had a significant impact on the staying time (*F* = 23.02, *p* < .001). More specifically, participants decided to stay 33.93 rounds with low expectancy, 60.03 rounds with moderate expectancy, and 83.55 rounds with high expectancy options. Differences in staying times between low and moderate expectancy options (*t* = 26.38, *p* < .001) and moderate versus high expectancy options (*t* = 27.17, *p* < .001) were significant. The main effect of task version on stay trials was not significant (*F* = 2.56, *p* = .110), however there was a significant interaction effect (Fig 5) of expectations by task version on staying times (*F* = 9.63, *p* < .001). Tukey’s HSD post-hoc tests revealed that participants who played the social version of the game decided to stay significantly (*z* = 9.67, *p* = .018) longer with moderate (60.77 rounds) rather than low (51.54 rounds) expectancy partners; and significantly (*z* = 14.15, *p* < .001) longer with high (72.65 rounds) rather than moderate expectancy partners. Participants who played the nonsocial version of the game stayed significantly (*z* = 43.74, *p* < .001) longer with moderate (69.12 rounds) rather than low (12.19 rounds) expectancy trees; and again significantly (*z* = 49.87, *p* < .001) longer with high (98.24 rounds) expectancy trees. Interestingly, participants stayed significantly (*z* = 36.51, *p* < .001) longer with social rather than nonsocial low expectancy options; and significantly (*z* = -33.28, *p* < .001) shorter with social rather than nonsocial high expectancy options. There was no difference in staying times between social and nonsocial moderate expectancy options (*z* = 2.45, *p* = .994). Overall, participants stayed longer with options the lower they estimated the performance of these options to be (*F* = 21.62, *p* < .001, *β* = -2.71), and this effect significantly interacted with task version (*F* = 47.06, *p* < .001).

In addition, participants stayed less with options the more high expectancy options they already had seen (*F* = 40.58, *p* < .001, *β* = -8.26); the fewer trials that were unsuccessful due to the option's failure (*F* = 13.57, *p* < .001, *β* = -4.23); and the more successful trials (*F* = 222.79, *p* < .001, *β* = 23.52). The percentage of trials that were unsuccessful due to failure by the participants themselves did not significantly impact performance estimations (*F* = 3.00, *p* = .090, *β* = 1.82).

### Performance estimations

#### The model

We assessed the degree to which participants’ estimations of their partners’ performance were accurate, based on the prior expectations they had about their partners. For this, we set up a mixed model (model 2B) with expectations, task version, and their interaction term as fixed factors; with the percentage of high expectancy options the participants had already been paired with, the percentage unsuccessful trials due to the 's failure, the participant's own failure, the number of successful trials; and we included a participant specific random intercept and random slopes for expectations.

#### Results

Results demonstrated a significant (*F* = 18.76, *p* < .001) effect of expectations on performance estimation. Participants estimated the low, moderate and high expectancy options to perform at, respectively, 31.69%, 45.08% and 60.88%. The difference in performance estimations between low and moderate expectancy (*t* = 13.73, *p* < .001), and between moderate and high expectancy (*F* = 15.68, *p* < .001) options were both significant. The effect of task version on performance estimations was also significant (*F* = 31.87, *p* < .001), such that, overall, participants estimated the trees (51.25%) to perform better than the partners (42.09%).

**Fig 6:**
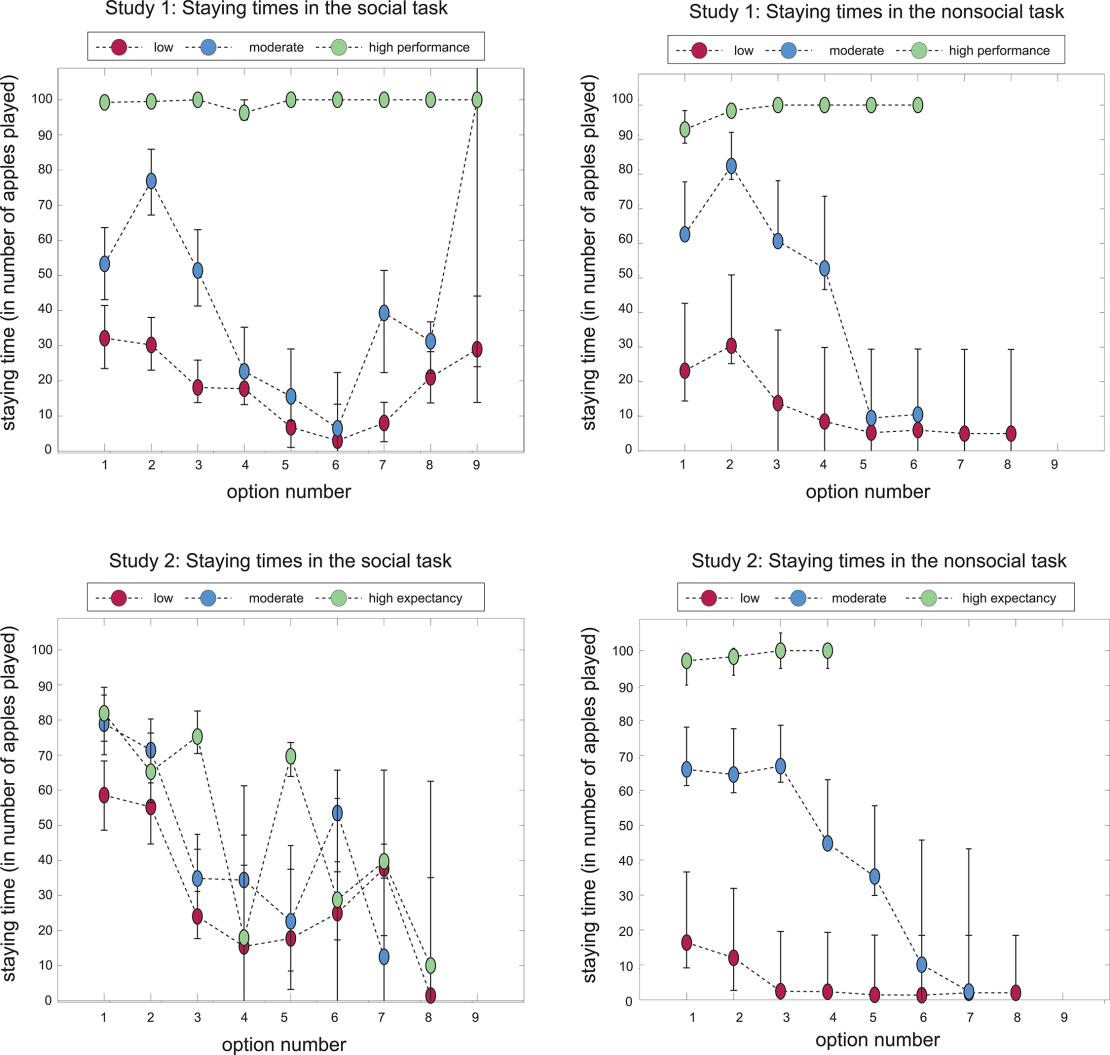
Staying times as a function of actual or expected performance levels in social and nonsocial task versions over time. In the top two subplots, staying times (y-axis) are plotted as a function of performance level (low, moderate or high; separate lines) and option number (i.e., the first low performing option, the second low performing option and etcetera). In the bottom two subplots, staying times (y-axis) are plotted as a function of prior expectations (low, moderate, high; separate lines) Note that the second low performing option may not have directly followed the first low performing partner; and that the first low-, moderate-, and high-performing s were not encountered at the same time. Error bars represent bootstrapped 95% confidence intervals.

Moreover, the interaction effect of expectations by task version on performance estimations (Fig 5) was also significant (*F* = 15.36, *p* < .001). Participants estimated the partners about whom they had low, moderate and high expectations to perform at, respectively, 36.72%, 42.33% and 47.10% (Fig 6), and the low, moderate and high expectancy trees were estimated to perform at, respectively, 25.48%, 48.48% and 79.46%.

Tukey’s HSD post-hoc tests revealed that participants did not estimate the moderate expectancy partners to perform significantly better than the low expectancy ones (*z* = -4.57, *p* = .148), but that the high expectancy partners were estimated as performing significantly better than the moderate expectancy ones (*z* = 6.17, *p* = .019). Also, participants estimated the moderate expectancy trees to perform significantly (*z* = 22.19, *p* < .001) better than the low expectancy ones; and the high expectancy trees to perform significantly (*z* = 32.12, *p* < .001) better than the moderate expectancy ones. As with the staying times, participants estimated the low expectancy partners to perform significantly (*z* = 10.88, *p* < .001) better than the low expectancy trees; and they estimated the high expectancy partners to perform significantly (*z* = —32.68, *p* < .001) worse than the high expectancy trees. There was no difference in performance estimations between moderate expectancy partners and trees (*z* = -6.73, *p* = .053).

In addition, performance estimations significantly increased the lower the percentage of trials on which participants lost points due to their option's failure (*F* = 11.91, *p* < .001, *β* = -2.33); the higher the percentage of trials on which participants lost points due to their own failure (*F* = 44.65, *p* < .001, *β* = 5.01); the higher the percentage of successful trials (*F* = 127.94, *p* < .001, *β* = 9.65); and the more high expectancy options the participants had already been paired up with (*F* = 5.16, *p* = .020, *β* = 1.59).

### Mediation analysis

Given the similarity of findings between the influence of prior expectations on staying times and on performance estimations, we performed post-hoc analyses to investigate whether the effect of prior expectations on staying times was mediated by participants’ performance estimations. To this end, we inserted the output of model 2A and model 2B into the mediate function of the mediation package in R. This revealed that 10.38 of the total increase of 11.55 apples that participants played with high rather than moderate expectancy partners were directly caused by our expectation induction (*p* = .00); and a remaining 1.17 apples were mediated (*p* = .00) through participants' performance estimations. Furthermore, of the 10.75 apples that participants played less with low rather than moderate expectancy partners, 9.40 were directly caused by our expectation induction (*p* = .00); and the remaining 1.17 apples were mediated through participants' performance estimations (*p* = .00). We can thus state that the effect of prior expectations on staying times was partially mediated by performance estimations.

### Differences between social and nonsocial condition

The simplest explanation for the finding that stay/leave decision making was less impacted by expectations in the social as compared to the nonsocial context could be that the prior expectation induction simply had a weaker effect on participants initial beliefs in the social than nonsocial condition. Unfortunately, our dataset does now allow us to investigate if and how the expected value of low-, moderate-, and high-expectancy options was different in social and nonsocial task at the very beginning of the task (i.e., trial 1) so we cannot rule out this explanation completely.

A more interesting explanation however would be that participants updated their prior beliefs differently in the social as compared to nonsocial task version. To investigate whether there is some indication that participants had different belief update functions across task versions, we plotted staying times as a function of expectations (low, moderate, high) and option number (i.e., first low-expectancy partner encountered, second low-expectancy partner encountered etc). Some practical issues should be noted: That is, participants varied considerably in the number of options they encountered in total (depending on their stay/leave decision making) so the higher the option number, the less observations we have for that number, and the greater the variance. Our plots indicate that staying times remained maximum for the high-expectancy options in the nonsocial task, whereas staying times decreased for the low-, and moderate-expectancy options in this same task. Interestingly, the pattern in the nonsocial task version, but not the social task version, is the same pattern of results that we see in Study 1 when *actual* performance levels were manipulated. This finding seems to illustrate that participants in the nonsocial task version treated our prior-expectation induction as if it were true information, and that this did not change once participants had encountered options that were either performing better or worse than expected. In contrast, the alternative pattern of results in the social task version suggests that in the social task version prior expectations were corrected for with participants' actual observations, especially for the high-expectancy option.

## Conclusion

Study 2 confirmed the hypothesis that prior expectations about our interaction partners have an important impact on subsequent stay/leave decisions. That is, if participants had low prior expectations about options, they gave up on them relatively quickly, whereas they stayed longer with high expectation options than might be expected given their actual performance. Interestingly, the effect of prior expectations on stay/leave decisions was much stronger in the nonsocial as compared to social task version. Even though all partners performed at chance levels, with no partner actually better or worse than any other, participants stayed 10 extra rounds with a partner if they had expected them to perform moderately as opposed to poorly. More dramatically, when participants expected a tree to perform badly, these trees were disposed with quickly (on average after just 12 rounds). In contrast, when participants expected a tree to perform well, they stayed 98 rounds with those trees. Importantly, this pattern was also evident in participants’ estimation of the options’ performance, and post-hoc analyses demonstrated that the effect of prior expectations on staying times was partially mediated by participants’ estimations of their options’ performance.

## Discussion

The aim of the present paper was to investigate whether decisions to stay with or leave social partners are based upon value-maximization motives, as suggested by some prior research [2, 22, 23], and additionally to explore how prior expectations about the value of partners affects decisions to either stay with or leave them during an ongoing collaborative task. Consistent with our hypothesis, we first demonstrated that participants decided to stay longer in collaborative partnerships when the probability of success of that partnership was high rather than low. Secondly, we demonstrated that, when the objective performance of all partners was actually equal, participants decided to stay longer with high-expectancy as compared to low-expectancy partners. In addition, our results demonstrated that even though prior expectations have a strong impact on stay/leave decisions in both social and nonsocial conditions, the effect was more pronounced in nonsocial conditions.

Existing literature has already shown a strong association between self-reported relationship satisfaction on the one hand, and self-reported relationship commitment, intended relationship commitment in hypothetical scenarios, and actual relationship maintenance (regardless of which partner made the decision to terminate the relationship) in real-life, on the other hand. However, to better understand the underlying processes of the *decision* to stay with or leave a social partner it is important that we investigate how the actual value that we derive from specific relationships affects stay/leave decision making about those relationships. Study 1 provides the first experimental evidence that actual stay/leave decision making is causally affected by relationship returns, at least in collaborative relationships, and potentially in a similar way as stay/leave decision making is affected by outcomes in nonsocial decision making contexts. This finding is important because it lays the foundation for future research on the mechanisms underlying actual social stay/leave decision making, such that it provides experimental support that social stay/leave decision making may be thought of in a framework of value-based decision making [24]. In addition, Study 1 demonstrated that the relationship between cooperation and stay/leave decision making is bidirectional. That is, while previous decision making studies demonstrates that having the option to leave a social partner increases cooperation levels [5, 6]; Study 1 now also demonstrated that the level of cooperation of a social partner also affects the decision to leave.

Study 2 demonstrated that prior beliefs about the value of our interaction partners also affects our decision to stay with or leave them; and specifically, that these beliefs bias decision making rather than serving as a frame of reference against which actual relationship returns are compared. In this way, the current findings are consistent with previous studies that showed that the presence of false priors can lead to maladaptive decision making in both nonsocial [16, 17, 25] and social [10, 18] dynamic decision making tasks.

A potential mechanism for this effect is that expectancy-consistent and expectancy-inconsistent observations about both social and nonsocial options’ successes and failures were differentially updated (i.e., a confirmation bias). Specifically, it could be that when participants update their beliefs about the value of specific options, they put more weight on behaviors of these options that were consistent with their prior beliefs than on behaviors that were inconsistent with those beliefs. In fact, related studies that demonstrated similar effects of prior beliefs on subsequent decision making did find support for such mechanism [10, 16–18, 25, 26]. By using computational modeling techniques, they found that the presence of prior expectations can lead to suboptimal decision making because it inhibits associative learning, and thus expectancy-consistent observations are weighted heavier than expectancy-inconsistent observations when people update prior beliefs about the probability of success of experimental stimuli, or the trustworthiness of a social partner with actual observations. An interesting avenue for future research is to use similar data analysis techniques to look further into how prior expectations affect the learning processes [7, 27] underlying decision making about social partners in a collaborative context. Unfortunately, performing these types of data analyses was not possible on the current data set.

Another interesting finding is that prior expectations did not just affect decision making per se, but also the value estimations underlying value-based decision-making. If, as in previous studies, participants also weighed expectancy-consistent observations more strongly than expectancy-inconsistent observations, it could be the case that participants considered successful trials by a high-expectancy partner as evidence for the belief that the partner was a productive one, while misses by this partner were dismissed (i.e., ‘everybody can make a mistake sometime’). Similarly, misses by a low-expectancy partner could then strengthen the belief that the partner was a poor one, while successful catches could be considered lucky shots. To investigate whether prior expectations actually biased participants’ perceptions of a relationship partner, participants were asked at the conclusion of each partnership to estimate the performance of the respective partners. Although the current study cannot demonstrate whether expectancy-inconsistent observations were selectively discarded, we did find that participants believed that the low expectancy partners caught fewer apples and the high expectancy partners caught more apples than other social partners, despite the absolute performance of all partners being identical. Additionally, mediation analyses demonstrated that the effect of prior beliefs on the time that participants stayed with their partners was partially mediated by participants’ (mis)perceptions of the efficacy of the various partners. The present findings that stay/leave decision making in social collaborations is based upon value-maximization, and that prior expectations bias both stay/leave decisions as well as beliefs about a person’s collaborative value, has important implications for real-life social relationships.

Extending previous studies that investigated the influence of prior beliefs on subsequent decision making, the present research directly compared the influence of prior expectations on decision-making between social and nonsocial contexts. We explored potential differences between social and nonsocial conditions as specific ‘social’ motivations such as bonding or guilt might have led participants to decide to stay longer with their social partners than with trees. It should be noted that, in the first study, we found no differences between social and nonsocial context, observing that participants stayed with social partners equally as long as with trees with similar performance levels. These findings suggests that motives of value-maximization affect decisions to stay or leave equally strongly across social and nonsocial contexts, with no evidence of social motivations modulating this effect between social and nonsocial context.

Nonetheless, we did find that the effect of prior expectations on stay/leave decisions was much less pronounced in a social than in a nonsocial context. Importantly, these results show that in a social stay/leave decision making context, participants were affected by prior expectations but did not follow them blindly. That is, if participants were simply doing as they were instructed (i.e., following the information about partner performance) they should have immediately left low expectancy partners rather than staying for over 50 rounds with those partners. The data demonstrates that they weighted both prior expectations and actual observations when deciding to stay or leave. In contrast, results demonstrate that participants were in fact more or less ‘blind’ to their actual observations in the nonsocial condition, where they seemed to follow our prior expectancy induction more closely. Given that the two tasks were designed to be objectively the same, even with respect to the monetary outcomes, it is interesting to find any actual differences between social and nonsocial contexts.

As mentioned earlier, a simple explanation for our findings is that our prior belief induction was less effective in social than nonsocial task. While we cannot completely rule out this explanation, our results do suggest that, at least, additional processes are occurring. That is, staying times for low-, moderate-, and high-expectancy options developed across the duration of the experiment in a similar fashion as staying times when actual performance levels were different (i.e. in Study 1). Specifically, in these conditions, staying times for high-performing and high-expectancy options remained maximum across the entire experiment, whereas staying times for less high-performing and low-, and moderate-expectancy options decreased over the course of the experiment. Interestingly, in the social task version of Study 2 we saw a different pattern of results. That is, staying times for the high-expectancy options decreased over the course of the experiment, as did staying times for low-, and moderate-expectancy options. These results suggest that, in the social condition alone, beliefs about the high-expectancy option at least, were corrected with the observation that the high-expectancy options were not as high-performing as they had been led to believe. One potential explanation for this differential effect of prior beliefs on decision making between social and nonsocial contexts could be that the relative strength of social priors is weaker than the strength of nonsocial priors, perhaps because social partners are less predictable than nonsocial events generally. Indeed, previous research demonstrates that learning rates are enhanced when there is increased uncertainty in the learning environment, at least in nonsocial contexts [28].

Another, potentially related, explanation is that people, implicitly, pay more attention to social than nonsocial information, perhaps because social information is more salient in general. Potentially then, this greater attentional focus in the social condition did not play a role in the Study 1 because participants had no prior knowledge in either condition about the partners or trees, and therefore had to actively pay attention to play the game optimally. In the second study however, prior knowledge about partners and trees was available, which may have lowered participants’ attention to their game partners’ performance. If indeed participants were implicitly more attuned to the performance of partners rather than trees, this implicit higher attentional focus may explain why the effect of prior expectations on stay/leave decisions was less pronounced in this social condition.

Finally, players were quite loyal to their game partners in the task, with many participants staying the full 100 trials with their partners. Reasons for this could be that the decision to stay was the default, with the decision to leave both consequential (you could not return to a previous partner) and ambiguous (there was no information provided on what the future might bring). Since the decision to leave was ambiguous–and people are commonly observed to be ambiguity-averse [29]–participants may have been hesitant to leave, and instead defaulted to the ‘stay’ option. Future versions of this task could endeavor to promote more leave decisions by, for example, giving participants information about the pool of alternative game partners, giving them the opportunity to return to a former game partner, or even making the decision to stay more difficult, for example by requiring participants to pay to stay. Acquiring a greater balance between stay and leave decisions would be especially important if investigating whether the effects of prior expectations on staying times are caused by a differential weighting of expectancy-consistent and inconsistent behavior of a collaborative partner. Notably, to perform these types of analyses the number of stay versus leave decisions should be relatively balanced, which was not the case in the current study.

## Conclusion

In conclusion, the present research demonstrated that (1) value maximization is indeed a fundamental aspect of stay/leave decision making in social (as well as non-social) contexts, and that (2) the prior beliefs we have about social partners guides subsequent stay/leave decisions, likely in a confirmatory manner. These findings are consistent with previous literature, but interestingly, the present paper directly compared the effect of prior beliefs on decision making and showed that (3) this latter effect was much less pronounced in a social than in a nonsocial context. It therefore seems that certain aspects of social relationships ‘protect’ people from the influence of prior expectations on social decision making in particular, offers interesting avenues for future research on this theoretically and practically important topic.

## Supporting Information

S1 Appendix Task instructions in the social condition of Study 1 and 2. (DOCX)

S2 Appendix Task instructions in the nonsocial condition of Study 1 and 2. (DOCX)

S3 Appendix Data Study 1. (CSV)

S4 Appendix Data Study 2. (CSV)
