# Supplementary material for: How Social and Nonsocial Context Affects Stay/Leave Decision-Making: The Influence of Actual and Expected Rewards
Source: PLoS One. 2015 Aug 7;10(8):e0135226. doi: 10.1371/journal.pone.0135226 (PMC4529303; doi:10.1371/journal.pone.0135226)
Supplement: S1 Appendix — (DOCX) [file pone.0135226.s001.docx]

INSTRUCTIONS APPLE GAME

Please read the following instructions for the Apple Game carefully. The Apple Game is a video game, which you will be playing simultaneously with other participants. The goal is to end with as many points as possible.

The money bonus

For every twenty participants, one will win a money bonus on top of the credit you will receive for participation. **Who** will receive the bonus, is **completely random**; we let the computer randomly select participant numbers at the end of the study who will receive the bonus. However, **the height** of the bonus is depends on your performance in the Apple Game. The higher the **amount of points** is you have at the end of the game, the higher the money bonus will be. Every point is worth 0.5 Eurocent.


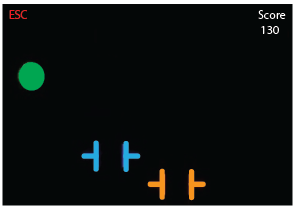
How to score points?

You score points by catching apples together with another participant (i.e., ***the game partner***). In the game, apples will fall from random locations from the top of the screen. It is your task to catch the apples by placing a tube (i.e., ***the basket***) under the apple, so that the apple will fall through the basket. The blue basket is always your basket; the orange basket always belongs to the game partner. You can move the blue basket by pressing the ***keys A (basket to left) and L (basket to right).*** At the same time, you will see your game partner do the same with his / her orange basket.

The score

For every apple that goes through both your baskets, you will both receive 10 points. For every apple that is missed by just you, just your game partner, or by the both of you, you will both lose 5 points. It could thus happen that you catch an apple while your game partner misses this apple. Even though you caught the apple, you will still lose 5 points. The same goes for your game partner of course.


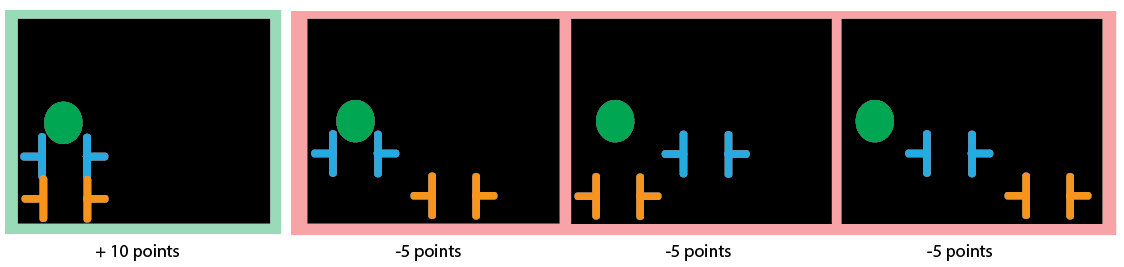


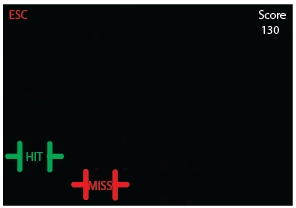
The feedback

When an apple reaches the bottom of the screen, you will receive feedback about who of you caught the apple and how many points you have now. First, you will see that the baskets change color. A basket turns green when an apple went through it; a basket turns red when the basket missed the apple. The words MISS and HIT are also displayed in the baskets. Moreover, you will see your score at the upper right corner of the screen change.

Escape

Since you are dependent of your game partner for the money bonus you might win, we implemented the following option: **You can indicate that you want a different game partner.** You do this by pressing **ESCAPE** on your key board. When you press ESC the game will be stopped immediately. You will be assigned to a random other game partner, whose performance may differ from your former game partner. You will then continue playing the game with your new game partner. **You can change partners whenever and as often as you like**. Important to know is that **your points** (as well as your game partner’s points) **are always preserved** when you change partners. Because there are more ‘orange-basket’ players than ‘blue-basket’ players, there will always be a game partner (with an orange basket) available. However, sometimes it can happen that a game is stopped even though you did not press ESC. When this happens, this just means that your partner is already finished with the study. You will be randomly assigned to a new game partner in case this happens.

The game partner

The game partner is a random other participant with whom you are coupled for a game. The game partners are other participants who signed in for a different study, and play at other locations in this and other labs at the university. Your computers are connected through a secure network. These players play the same Apple Game, with the only differences being that they move the orange basket; and that **they cannot leave their game partners.**

Introducing the game partners (Study 2 only)

Every time when you are assigned to a new game partner, you will receive a short introduction of your new game partner. In this introduction you will receive some information that indicates how good your new partner was in his /her previous games.

Second, you will see an indication of **how many apples your new partner caught** in their previous game (regardless of what his/her partners did). How good a new game partner is will be indicated by stars.
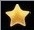
1 Star means that this game partner caught **less than half** of the apples (0 – 33%);
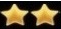
 2 stars means that this game partner caught **about half** of the apples (34% - 66%);
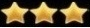
 3 stars means that this game partner caught **more than half** of the apples (67% - 100%). So the stars are an indication of how good the partner was in the past game, but this does not necessarily mean anything for his / her future games: People may vary in how good they are in catching apples.

Estimation


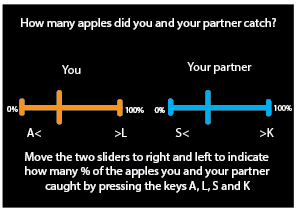
At the end of each game (so every time you will receive a new partner), the computer will ask you to **estimate the percentage of apples you** (regardless of your game partner) **and your game partner** (regardless of you) **caught** in the previous game. You will see two bars ranging from 0% to 100% on which you can move a marker. As you can see in the screenshot, you can move the markers by pressing the keys A and L (for your percentage) and S and K (for your partners’ percentage). Only when you **press SPACE**, your estimation will be confirmed and the game (with a new partner) will be continued). Please remember that this is estimation, it does not matter if you are not exactly right.

*Please open the door of your cubicle now, so that the researcher can see that you finished reading the instructions. If you have any questions, you can ask them to her.*
