# Supplementary material for: How Social and Nonsocial Context Affects Stay/Leave Decision-Making: The Influence of Actual and Expected Rewards
Source: PLoS One. 2015 Aug 7;10(8):e0135226. doi: 10.1371/journal.pone.0135226 (PMC4529303; doi:10.1371/journal.pone.0135226)
Supplement: S2 Appendix — (DOCX) [file pone.0135226.s002.docx]

INSTRUCTIONS APPLE GAME

Please read the following instructions for the Apple Game carefully. The Apple Game is a video game, in which the goal is to end with as many points as possible.

The money bonus

For every twenty participants, one will win a money bonus on top of the credit you will receive for participation. **Who** will receive the bonus, is **completely random**; we let the computer randomly select participant numbers at the end of the study who will receive the bonus. However, **the height** of the bonus is depends on your performance in the Apple Game. The higher the **amount of points** is you have at the end of the game, the higher the money bonus will be. Every point is worth 0.5 Eurocent.


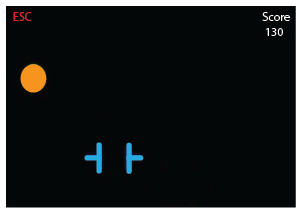
How to score points?

You score points by catching apples that will fall from random locations from the top of the screen. It is your task to catch the apples by placing a tube (i.e., ***the basket***) under the apple, so that the apple will fall through the basket. You can move the basket by pressing the ***keys A (basket to left) and L (basket to right).***

The score


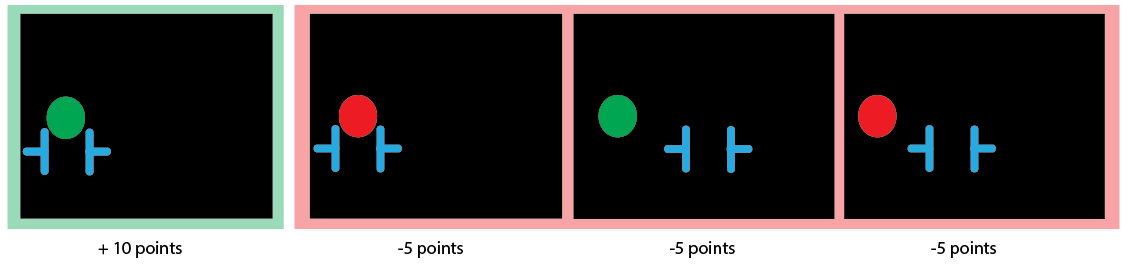
There is an extra feature of the game that is important for your money bonus. That is, some of the apples that fall are ripe and some are unripe. For every ripe apple that goes through your basket, you will receive 10 points. For every apple that is missed by you, that is unripe, or that is both missed and unripe, you will lose 5 points. It could thus happen that you catch an apple that turns out to be unripe. Even though you caught the apple, you will still lose 5 points.


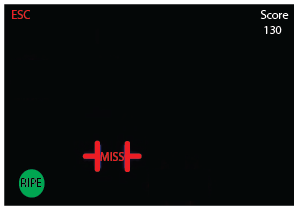
The feedback

When an apple reaches the bottom of the screen, you will receive feedback about whether you caught the apple and how many points you have now. First, you will see that the basket changes color. The basket turns green when an apple went through it; the basket turns red when the basket missed the apple. The words MISS and HIT are also displayed in the basket. Moreover, you will see your score at the upper right corner of the screen change.

Escape

Since your money bonus depends also on the number of ripe and unripe apples a specific tree drops, we implemented the following option: **You can indicate that you want a different tree.** You do this by pressing **ESCAPE** on your key board. When you press ESC the game will be stopped immediately. You will be assigned to a random other tree of which the percentage of ripe apples dropped may differ from that of your former tree. You will then continue playing the game with your new tree. **You can change trees whenever and as often as you like**. Important to know is that **your points are always preserved** when you change trees. There is always a new tree available. Sometimes it can happen that a tree has no more apples left. In this case you will also be assigned to a different tree.

Introducing the trees (Study 2 only)

Every time when you are assigned to a new tree, you will receive a short introduction of your new game tree. That is, you will see an indication of **how many ripe apples your new tree** dropped when previous participants were assigned to it. How many ripe apples a tree dropped will be indicated by stars.
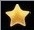
1 Star means that **less than half** of the apples this tree dropped were ripe (0 – 33%);
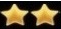
 2 stars means that **about half** of the apples this tree dropped were ripe (34% - 66%);
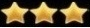
 3 stars means **more than half** of the apples this tree dropped were ripe (67% - 100%). So the stars are an indication of how many ripe apples the tree dropped in the past, but this does not necessarily mean anything for the future. Trees may vary in how many ripe apples they drop.


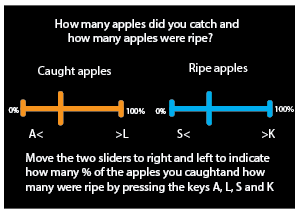
Estimation

At the end of each sub game (so every time you will receive a tree), the computer will ask you to **estimate the percentage of apples you** **caught** (regardless of whether these were ripe) and how many ripe apples the former tree had dropped (regardless of whether you caught them) in the previous game. You will see two bars ranging from 0% to 100% on which you can move a marker. As you can see in the screenshot, you can move the markers by pressing the keys A and L (for your percentage) and S and K (for the tree’s percentage). Only when you **press SPACE**, your estimation will be confirmed and the game (with a new tree) will be continued. Please remember that this is estimation, it does not matter if you are not exactly right.

*Please open the door of your cubicle now, so that the researcher can see that you finished reading the instructions. If you have any questions, you can ask them to her.*
